# Complex *MSH2* and *MSH6* mutations in hypermutated microsatellite unstable advanced prostate cancer

**DOI:** 10.1038/ncomms5988

**Published:** 2014-09-25

**Authors:** Colin C. Pritchard, Colm Morrissey, Akash Kumar, Xiaotun Zhang, Christina Smith, Ilsa Coleman, Stephen J. Salipante, Jennifer Milbank, Ming Yu, William M. Grady, Jonathan F. Tait, Eva Corey, Robert L. Vessella, Tom Walsh, Jay Shendure, Peter S. Nelson

**Affiliations:** 1 Department of Laboratory Medicine, University of Washington, Seattle, Washington 98195, USA; 2 Department of Urology, University of Washington, Seattle, Washington 98195, USA; 3 Department of Genome Sciences, University of Washington, Seattle, Washington 98195, USA; 4 Division of Human Biology, Fred Hutchinson Cancer Research Center, Seattle, Washington 98109, USA; 5 Division of Clinical Research, Fred Hutchinson Cancer Research Center, Seattle, Washington 98109, USA; 6 Division of Medical Genetics, Department of Medicine, University of Washington, Seattle, Washington 98195, USA

## Abstract

A hypermutated subtype of advanced prostate cancer was recently described, but prevalence and mechanisms have not been well-characterized. Here we find that 12% (7 of 60) of advanced prostate cancers are hypermutated, and that all hypermutated cancers have mismatch repair gene mutations and microsatellite instability (MSI). Mutations are frequently complex *MSH2* or *MSH6* structural rearrangements rather than *MLH1* epigenetic silencing. Our findings identify parallels and differences in the mechanisms of hypermutation in prostate cancer compared with other MSI-associated cancers.

## Abstract (web summary)

Several patients with metastatic prostate cancer have been shown to harbour tumours with markedly high mutation rates. Here, the authors characterise hypermutation in advanced prostate cancer samples and show that these samples have somatic mismatch repair gene mutations and microsatellite instability.

## Graphical abstract

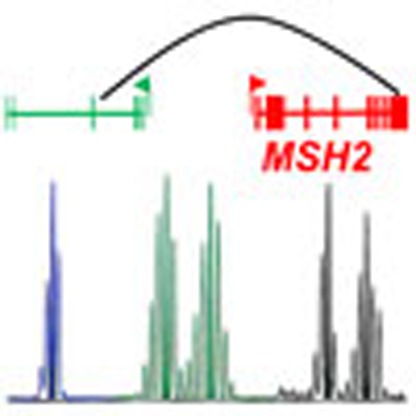

Recently exome sequencing of metastatic prostate cancers revealed that a subset of patients harboured tumors with markedly elevated single-nucleotide mutation rates, defining a new hypermutated subtype^1^. This phenotype was subsequently observed in primary prostate cancer in a tumour that harboured an *MSH6* mutation^2^. However, mechanisms that lead to hypermutation and the prevalence of this distinct subtype have not been completely defined. Comprehensive cancer genomics efforts recently published by The Cancer Genome Atlas Research Network (TCGA) reported that 16% of colon cancers and up to 35% of endometrial cancers exhibit hypermutation^3^,^4^. For both colon and endometrial cancers, about three quarters of hypermutated tumors were associated with phenotypic microsatellite instability (MSI) and loss-of-function DNA mismatch repair genes via mutation or epigenetic silencing. Therefore, we hypothesized that hypermutated prostate cancer may also be associated with DNA mismatch repair (MMR) gene defects and MSI.

In this study we identified hypermutation in 7 of 60 patients with advanced prostate cancer. Using a targeted deep sequencing approach we find that all hypermutated tumors have somatic mutations in MMR genes and associated MSI. In four of seven hypermutated cases MMR mutations were complex structural rearrangements in *MSH2* and *MSH6*. We conclude that somatic rearrangements in *MSH2* and *MSH6* are an important mechanism leading to hypermutation and MSI in advanced prostate cancer.

## Results

### Prevalence of hypermutation

We identified hypermutated cases in exome sequencing data sets of advanced prostate cancer samples from two sources: a panel of patient-derived xenografts (PDX) and metastatic specimens obtained through a rapid autopsy programme (Supplementary Table 1). Exome data for PDX tumors was from Kumar *et al*.^1^, where hypermutation was previously characterized. In the autopsy samples where hypermutation status had not been previously established, we defined hypermutation as >300 somatic protein altering mutations based on the distribution of total mutation burden in metastatic tumors, which had matched normal tissue available (Supplementary Fig. 1; Supplementary Table 1). We identified hypermutation in 3 of 15 PDX tumors (Table 1), and in metastatic tumors from 5 of 50 autopsy patients (Table 2). There was partial overlap between the two patient groups: five of the PDX tumors were derived from autopsy patients, including one with a hypermutated genome (LuCaP 147). Therefore, there were a total of 7/60 unique patients with hypermutated tumors, for an overall prevalence of 11.6%. Hypermutation status was 100% concordant at different metastatic sites, and was also concordant between primary tumour and metastasis in two patients where primary prostate tumors were available (Table 2).

### Identification of *MSH*2 and *MSH6* rearrangements

Because exome sequencing has limitations in detecting structural rearrangements and larger insertion/deletion (indel) mutations, we investigated alterations in DNA MMR pathway genes in hypermutated and non-hypermutated cases using a targeted deep sequencing approach (BROCA assay) that included capture of intronic and flanking DNA sequences (Supplementary Table 2)^5^,^6^. We developed a bioinformatics pipeline to accurately detect structural variation, copy number variation and indel mutations of all sizes^7^.

**Figure 1:**
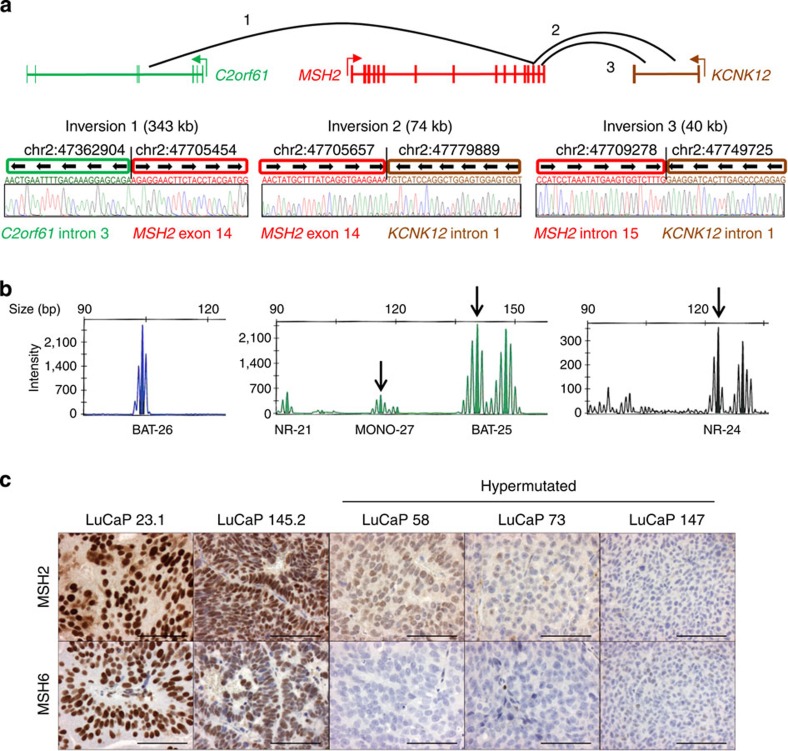
*MSH2* and *MSH6* rearrangements are associated with loss of protein expression and MSI. (**a**) Four of seven hypermutated cases had complex rearrangements in *MSH2* and *MSH6* or both genes. Shown is a representative complex *MSH2* rearrangement present in hypermutated cases LuCaP 147 and 05–165 (LuCaP 147 was derived from autopsy patient 05–165). Breakpoints were confirmed by Sanger sequencing. Genomic coordinates are hg19. Detail on additional structural rearrangements and other mismatch repair gene mutations is provided in Tables 1 and 2 and Supplementary Figs 2–9. (**b**) Hypermutated tumors exhibited microsatellite instability by PCR. Shown is representative data for LuCaP 58, which is positive for MSI in 3/5 mononucleotide marker systems (MONO-27, BAT-25 and NR-24, arrows). All hypermutated tumors tested were MSI-PCR positive in at least 2/5 loci (Supplementary Data 1). (**c**) Hypermutated tumors LuCaP 58, 73 and 147 have loss of MSH2 and MSH6 proteins by IHC. Similar results were observed in hypermutated tumors from rapid autopsy patients (Supplementary Fig. 11). A representative non-hypermutated tumour (LuCaP 23.1) has intact expression. LuCaP 145 had mono-allelic mutations in *MSH2* and *MSH6* but was not hypermutated. IHC shows loss of MSH6 protein expression in some tumour cells. Scale bars, 0.1 mm.

All three PDX hypermutated tumors had complex structural rearrangements in *MSH2*, *MSH6* or both genes (Table 1; Fig. 1a; Supplementary Figs 2–4), while only 1 of 20 non-hypermutated xenografts had mutations in these genes (LuCaP 145, derived from a patient with neuroendocrine prostate cancer, Supplementary Fig. 5). A second loss-of-function mutation in *MSH2* or *MSH6* was detected in the three hypermutated PDX tumors, but not in LuCaP 145, supporting a requirement for bi-allelic gene inactivation underlying the hypermutated genome.

We detected mutations with predicted loss-of-function in *MSH2*, *MSH6* or both genes in four of five rapid autopsy patients with hypermutated tumors. Mutations included complex structural rearrangements, copy losses and frameshift mutations (Table 2; Supplementary Figs 4 and 6–9). Two hypermutated patients had mutations in the MMR gene *MLH1*. We interrogated a subset of six non-hypermutated patients by deep sequencing and did not detect MMR gene mutations except in patient 05–144 from which the PDX LuCaP 145 was derived (Table 2). Like hypermutation status, MMR mutations were concordant at different metastatic sites in the same patient. MMR mutations were also concordant between primary tumour and metastasis except for a single *MLH1* frameshift mutation in patient 05–123 not found in the primary tumour (Table 2; Supplementary Fig. 9). Patient-matched non-tumour tissues were tested for the autopsy patients (Supplementary Table 1 and Supplementary Data 1). No MMR mutations were detected in patient-matched non-tumour tissue, indicating that none of the MMR mutations were inherited in the germline. Mutations in additional DNA repair genes are given in Supplementary Table 3.

### Hypermutated tumors have phenotypic MSI

*MSH2* and *MSH6* are mismatch DNA repair genes that act together as a heterodimer, and bi-allelic inactivating mutations of either gene are predicted to result in MSI. PCR of microsatellite loci revealed MSI in all hypermutated tumors, from both PDX and autopsy patients (Fig. 1b; Supplementary Data 1). Phenotypic MSI was also detected directly from targeted next-generation data for all hypermutated tumors, and not detected in any non-hypermutated tumors (Supplementary Data 1; Supplementary Fig. 10). Immunohistochemistry (IHC) for DNA MMR proteins in hypermutated tumors demonstrated complete loss of MSH2 and/or MSH6 in a pattern consistent with the inactivating mutations detected by sequencing (Fig. 1c; Supplementary Fig. 11). Non-hypermutated tumors were microsatellite stable (Tables 1 and 2; Supplementary Data 1) and had intact MSH2 and MSH6 proteins, except LuCaP 145, which exhibited heterogeneous loss of MSH6 protein (Fig. 1c). *MLH1* methylation was not detected in any of the MSI positive tumors (Supplementary Fig. 12), and MLH1 protein expression was intact by IHC in MSI-positive tumors except in 06–134 that had homozygous *MLH1* gene deletion (Supplementary Fig. 13), arguing that *MLH1* epigenetic silencing was not responsible for MSI in any of the tumors in our series.

## Discussion

Our findings support the conclusion that the hypermutated subtype of prostate cancer is chiefly due to loss-of-function mutations in *MSH2* and *MSH6* that result in MSI. Mutations were predicted to be bi-allelic in all cases except 00–010, which may harbour a second undetected mutation. Most interestingly, four of seven hypermutated cases had complex structural rearrangements in *MSH2* and *MSH6* that were not detected by exome sequencing in the same samples, and would also not be expected to be detected by traditional exon-based Sanger sequencing methods. Several previous studies have reported MMR protein loss and MSI in both primary and advanced prostate cancers, but very few MMR mutations have been identified^8^,^9^,^10^,^11^,^12^,^13^,^14^,^15^. We speculate that technical limitations have led to an underestimation of MMR gene mutations in prostate cancer.

Our finding of predominantly *MSH2* and *MSH6* mutations is in contrast to colon and endometrial cancer, where MSI is most often due to *MLH1* epigenetic silencing^3^,^4^. This supports an alternate mechanism by which MSI is acquired in prostate cancer. A recent study demonstrated that DNA translocations and deletions in advanced prostate cancer occur in a highly interdependent manner, a process termed ‘chromoplexy’^16^. This process may play a role in the genesis of *MSH2* and *MSH6* structural rearrangements and deserves future study. Androgen receptor (AR) function may also play a role in the formation of *MSH2* and *MSH6* structural alterations. AR has recently been implicated in the genesis of gene rearrangements in prostate cancer by facilitating double-strand DNA breaks and inducing non-homologous end-joining (reviewed in refs 17, 18).

In summary, we have shown that complex structural rearrangements in mismatch DNA repair genes *MSH2* and *MSH6* are a major mechanism underlying hypermutation in advanced prostate cancer. Future studies should focus on determining if patients with MMR gene defects exhibit a distinct clinical course and are differentially responsive to genotoxic therapy.

## Methods

### Patients and specimens

The LuCaP series of prostate cancer xenografts were obtained from the University of Washington Prostate Cancer Biorepository.

Human primary and metastatic prostate cancer tissues were obtained as part of the University of Washington Prostate Cancer Donor Rapid Autopsy Programme. A haematoxylin and eosin slide was reviewed and scrolls from tissue blocks with >50% estimated tumour purity were used. The Institutional Review Board of the University of Washington approved all procedures involving human subjects, and all subjects signed written informed consent. The sample size was chosen based on the number of cases with suitable tissues for exome sequencing.

Genomic DNA was prepared from either formalin-fixed paraffin-embedded tissue or from fresh-frozen tissue (for bone metastases) with the Gentra Puregene DNA Isolation Kit (Qiagen, Catalogue #158489).

### Immunohistochemistry

Expression of MMR proteins was determined by IHC using a tissue microarray (UWTMA55), that consisted of 155 metastatic prostate cancer sites from 50 patients, including 77 soft tissue metastases and 83 bone metastases), UWTMA52 consisting of primary prostate cancer obtained at the time of radical prostatectomy from 127 patients, and UWTMA 63 that consisted of prostate cancer tissue from 32 different LuCaP xenograft lines. All the tissue cores were duplicated.

Formalin-fixed paraffin-embedded tissue sections (5 μm) were deparaffinized and rehydrated with three changes of xylene and graded ethanol. Antigen retrieval was performed with heat-induced epitope retrieval for 20 min. Endogenous peroxide and avidin/biotin was blocked and sections were then blocked with 5% normal goat-horse-chicken serum at room temperature for 1 h, and incubated with primary antibody (listed in table below) at 4 °C overnight. After washing three times with 1 × PBS, slides were incubated with biotinylated secondary antibody (Vector Laboratories Inc.), followed by ABC reagent (Vector Laboratories Inc.) and stable diaminobenzidine (Invitrogen Corp.). All sections were lightly counterstained with haematoxylin and mounted with Cytoseal XYL (Richard Allan Scientific). Mouse or rabbit immunoglobulin-G was used at the same concentration as the primary antibody for negative controls. Antibodies and dilutions used for IHC are given in Supplementary Table 4.

Immunostaining was assessed using a quasi-continuous score system, created by multiplying each intensity level (‘0’ for no brown colour, ‘1’ for faint and fine brown chromogen deposition and ‘2’ for clear and coarse granular chromogen clumps) with the corresponding percentage of cells expressing the particular intensity, and then summing all values to get a final score for each sample (scores ranging from 0 to 200). Only nuclear staining was evaluated. Samples with damaged tissue core, missing tissue core or poor quality of tissue were excluded from finial analysis.

### Microsatellite instability PCR

MSI-PCR testing was performed by the University of Washington (UW) clinical genetics and solid tumors laboratory using the Promega MSI analysis kit (Promega, Madison, WI, USA) following the manufacturer’s instructions. Specimens demonstrating instability within two or more of the five mononucleotide markers included in this panel were considered ‘MSI positive’, others were considered ‘MSI negative’. The microsatellite loci tested in the Promega MSI analysis kit were NR-21, BAT-26, BAT-25, NR-24 and MONO-27 (Genbank Accession # XM_033393, U41210, L04143, X60152, AC007684, respectively).

### *MLH1* methylation analysis

Two to four hundred nanograms of DNA from each sample was bisulfite converted using the EZ DNA Methylation Kit (Zymo Research, Irvine, CA, USA) and eluted in 20 μl volume, according to manufacturer’s protocol.

SYBR Green qPCR to detect methylated and unmethylated *MLH1* was performed using a CFX 96 Touch Real-Time PCR Detection System (Bio-Rad, Hercules, CA, USA) with a final reaction volume of 20 μl, consisting of 500 nM each primer, 9 ng of bisulfite-converted genomic DNA and iTaq Universal SYBR Green Supermix at the following conditions: 95 °C for 3.5 min, followed by 40 cycles at 95 °C for 5 s and 60 °C for 30 s. The unique primer sequences for methylated *MLH1* were 5′- CGGATAGCGATTTTTAACGC -3′ (forward) and 5′- CCTAAAACGACTACTACCCG -3′ (reverse), and for unmethylated *MLH1* were 5′- AATGAATTAATAGGAAGAGTGGATAGT -3′ (forward) and 5′- TCTCTTCATCCCTCCCTAAAACA -3′ (reverse) (ref. 19). The four primers each also included a 20 bp GC-rich tail (5′- GCGGTCCCAAAAGGGTCAGT -3′) at their 5′ end. Repetitive Alu sequence (‘AluC4’) was used to normalize for the amount of input DNA2. The absolute quantitation of methylated and unmethylated *MLH1* in each sample was determined by using the Epitect human methylated and unmethylated DNA (Qiagen, Germantown, MD, USA) to create a standard curve. The SYBR Green assay results are expressed as ratios between methyl-*MLH1* or unmethyl-*MLH1* values and the ALUC4 control values. The error bars represent the s.e.m.

### Exome sequencing

Exome sequencing for autopsy samples was performed using the Nimblegen EZ SeqCap kit (Roche)^1^,^20^. Shotgun libraries were constructed by shearing DNA and ligating sequencing adaptors. Libraries were hybridized to either the EZSeqCap V1 or V2 solution-based probe, amplified and sequenced on either the Illumina GAIIx or HiSeq platform. For all metastases, somatic mutations were called using Mutect using default parameters with matched normal (non-tumour) samples. To remove common polymorphisms and other artifacts, we imposed a number of additional requirements, including requiring variants to be observed with a variant allele fraction of at least 10% within a tumour, removing variants present within dbSNP v137 that had first been stripped of all disease-associated variants and removing variants that were present at an allele balance of 40% or more in any germline sample. All exome sequencing was performed on fresh-frozen tissue samples.

Exome data for PDX samples was from Kumar *et al*.^1^, where hypermutation status was previously characterized based on the distribution of mutations across samples. For the xenografts, because corresponding normal germline DNA was not available, tumour sequences were compared against a database of common germline variants. The variants remaining were termed novel single-nucleotide variants SNVs (‘novSNV’) and the estimated the contribution of germline variants was ~200 and sometimes more per individual. novSNV counts from Kumar *et al*.^1^ are provided in Supplementary Table 1.

### Targeted deep sequencing by BROCA

Targeted deep sequencing of DNA repair pathway genes was performed using the BROCA assay in the UW clinical genetics and solid tumors laboratory^5^. Three micrograms of DNA was sonicated to a peak of 200 bp on a Covaris S2 instrument (Covaris, Woburn, MA, USA). Following sonication, DNA was purified with AMPure XP beads (Beckman Coulter, Brea CA, USA) and subjected to three enzymatic steps: end repair, A-tailing and ligation to Illumina paired-end adaptors as described in the SureSelectXT Target Enrichment for Illumina multiplexed sequencing, which is available for free download. Adapter-ligated library was PCR amplified for five cycles with Illumina primers 1.0 and 2.0 and individual paired-end libraries (500 ng) were hybridized to a custom design of complementary RNA biotinylated oligonucleotides targeting 53 genes in 52 genomic regions (Supplementary Table 2). The 120-mer oligonucleotide baits were designed in Agilent’s eArray web portal with the following parameters: centred tiling, 3 × bait overlap and a maximum overlap of 20 bp into repetitive regions. The custom design targets a total of 1.4 Mb of DNA. Following capture, each library was PCR amplified for 13 cycles with primers containing a unique 6 bp index. Equimolar concentrations of 96 libraries were pooled to a final concentration of 10 pM, denatured with 3 N NaOH, and cluster amplified with a cBot instrument on a single lane of an Illumina v3 flowcell. Sequencing was performed with 2 × 101 bp paired‐end reads and a 7 bp index read using SBS v3 chemistry on a HiSeq2500 (Illumina, SanDiego, CA, USA).

We used our targeted tumour sequencing bioinformatics pipeline for data analysis^21^. Reads were mapped to human reference genome (hg19/GRCh37) and alignment performed using BWA v0.6.1-r10419 and SAMtools v0.1.1820. SNV and indel calling was performed through the GATK Universal Genotyper using default parameters and using VarScan v2.3.2 and PINDEL version 0.2.42. Structural variants were identified using CREST v1.0 and BreakDancer v1.1. For copy number variant (CNV) analysis, copy number states for individual probes were initially called using CONTRA v2.0.32 with reference to a CNV control comprised of reads from two independent rounds of library preparation and sequencing of HapMap individual NA12878. CNV calls were made at the resolution of individual exons using custom Perl scripts. CNV plots were visualized using the R package ggplot2.

Phenotypic MSI was assessed directly from BROCA next-generation sequencing data using mSINGS (MSI by NGS)^22^. This method evaluated up to 146 mononucleotide microsatellite loci that are captured by BROCA in both matched normal non-tumour and tumour samples. For each specimen, microsatellite loci covered by a read depth of <30 × were excluded as not passing quality filter. For each microsatellite locus passing quality filter, the distribution of size lengths were compared with a population of normal controls. Loci were considered unstable if the number of repeats is statistically greater than in the control population. A fraction of >0.20 (20% unstable loci) was considered MSI-high by mSINGS based on validation with 324 tumour specimens, in which 108 cases had MSI-PCR data available as a gold standard^22^.

### Confirmation of *MSH2* and *MSH6* structural rearrangements

To validate structural rearrangement calls, we designed primers against regions flanking putative breakpoints using either PrimerBlast (http://www.ncbi.nlm.nih.gov/tools/primer-blast/) or Primer3 (http://bioinfo.ut.ee/primer3-0.4.0/primer3/input.htm). We used the iProof High-Fidelity PCR kit (Bio-Rad) to perform PCR under the following conditions: 98 °C for 35 s followed by 30–40 cycles of 55–69 °C for 30 s, 72 °C for 30 s and 72 °C for 10 min. Primers are listed in Supplementary Table 5. We submitted resulting PCR products to Genewiz for Sanger sequencing and aligned fragments to the human genome reference sequence (hg19) using BLAT from the UCSC Genome Browser (http://genome.ucsc.edu/cgi-bin/hgGateway).

Copy number changes were confirmed by genomic microarray. One microgram of high molecular weight genomic DNA from each sample was labelled by random priming using the Agilent Genomic DNA Enzymatic Labelling Kit (Cy3-dUTP.) A pool of reference normal DNA (Promega) was labelled with Cy5-dUTP. Cy3 and Cy5 probes were combined and hybridized to Agilent 2 × 400K SurePrint G3 CGH Microarrays and washed following the manufacturer’s specifications. Fluorescent array images were collected using the Agilent DNA microarray scanner G2505C and Agilent Feature Extraction software. Data analysis was performed with Biodiscovery Nexus Copy Number 6.0 software. The FASST2 segmentation algorithm and default Agilent settings for significance, gain and loss thresholds, with at least six probes per segment were used to identify regions of CNV for each sample. Results of copy number analysis by genomic microarray are given in Supplementary Fig. 14.

## Additional information

**How to cite this article:** Pritchard, C. C. *et al*. Complex *MSH2* and *MSH6* mutations in hypermutated microsatellite unstable advanced prostate cancer. *Nat. Commun.* 5:4988 doi: 10.1038/ncomms5988 (2014).

**Accession codes**: Sequencing data reported in this manuscript have been deposited in GenBank/EMBL/DDBJ under the accession code SRP044943.

## Supplementary Material

Supplementary Information Supplementary Figures 1-14, Supplementary Tables 1-6

Supplementary Dataset 1 Detail on BROCA and MSI Analysis

## Figures and Tables

**Table 1:** MMR gene mutations in prostate cancer PDX.

**PDX tumour** *	**Patient-derived from**	**Hypermutated?** †	**MSI**	**MMR gene mutation(s)** ‡
LuCaP 58		Yes	Yes	(1) *MSH6* del exon 8 to 3′UTR
				(2) *MSH6* frameshift (c.3799_3800del)
LuCaP 73		Yes	Yes	(1) *MSH2* and *MSH6* copy loss (del 3 Mb)
				(2) *MSH2*-*FBXO11* inversion
LuCaP 147, 147CR	05–165	Yes	Yes	(1) *MSH2*-*C2orf61* 343 kb inversion
				(2) *MSH2*-*KCNK12* 74 kb inversion
				(3) *MSH2-KCNK12* 40 kb inversion
LuCaP 23.1, 23.1CR		No	No	None
LuCaP 35, 35CR		No	No	None
LuCaP 70, 70CR		No	No	None
LuCaP 77, 77CR		No	No	None
LuCaP 78	98–328	No	No	None
LuCaP 81	98–362	No	No	Chr2 copy losses
LuCaP 86.2, 86.2CR		No	No	None
LuCaP 92	99–069	No	No	None
LuCaP 96, 96CR		No	No	None
LuCaP 105, 105CR		No	No	None
LuCaP 141		No	No	None
LuCaP 145.1, 145.2	05–144	No	No	(1) *MSH2* exon 8–16 del
				(2) *MSH6-TESC* t(2;12)

MMR, mismatch repair; MSI, microsatellite instability; PDX, patient-derived xenografts.

^*^Matched pairs of androgen-sensitive and castration-resistant sublines (for example, LuCaP 35 and LuCaP 35CR) and tumour lines derived from the same patient are listed numerically and grouped in the same row.

^†^Hypermutation status was previously determined in these samples in Kumar *et al*.^1^

^‡^Mosaic *MSH6* frameshift mutations observed in a poly G tract in exon 5 (c.3261dup/del) and poly A tract in exon 7 (c.3573del) were detected in several hypermutated samples and are not included in the table because they are presumed to be due to MSI.

**Table 2:** MMR gene mutations in rapid autopsy patients.

**Autopsy patient** *	**Tumour site(s) tested by BROCA targeted sequencing**	**Mutation burden**† **(exome)**	**Hypermutated?**	**MSI**	**MMR gene mutation(s)** ‡
05–165*	Bone, adrenal, liver and lymph node	855	Yes	Yes	(1) *MSH2*-*C2orf61* 343 kb inversion
					(2) *MSH2*-*KCNK12* 74 kb inversion
					(3) *MSH2-KCNK12* 40 kb inversion
03–130	Lymph node	647	Yes	Yes	(1) *MSH2* translocation splits the gene t(2;18)
					(2) *MSH2* copy loss
					(3) *MSH6* frameshift (c.2690del)
					(4) *MSH6* copy loss
06–134	Kidney and lymph node	314	Yes	Yes	*MLH1* homozygous copy loss
00–010	Prostate and liver	673	Yes	Yes	*MSH2* frameshift (c.2364_2365insTACA)
05–123	Prostate and lymph node	807	Yes	Yes	(1) *MSH2* frameshift (c.1124_1125insG)
					(2) *MSH2* frameshift (c.1082del)
					(3) *MLH1* frameshift (c.1310del), lymph node only
01–095	Liver and lymph node	149	No	No	None
05–144*	Bone, adrenal, liver and lymph node	57	No	No	(1) *MSH2* exon 8–16 del
					(2) *MSH6-TESC* t(2;12)
05–214	Bone, liver and lymph node (two sites)	46	No	No	None
05–116	Bone, adrenal, liver and lung	47	No	No	None
00–029	Liver	37	No	No	None
00–090	Lymph node	69	No	No	None

MMR, mismatch repair; MSI, microsatellite instability.

^*^Fifty total unique autopsy patients were assessed by exome sequencing (see Supplementary Table 1). Listed are a subset of cases that were followed up by targeted deep sequencing for MMR genes. Clinical data for this patient subset is provided in Supplementary Table 6. Patient-matched non-cancer tissue was tested in every case and did not exhibit MSI or MMR mutations. LuCaP 147 and 147CR are derived from autopsy patient 05–165. LuCaP 145.1 and 145.2 are derived from autopsy patient 05–144.

^†^Number of protein altering somatic mutations by exome sequencing with removing of germline variants from matched-non-tumour samples.

^‡^Mutations were detected at every tumour site unless otherwise indicated. Mosaic *MSH6* frameshift mutations observed in a poly G tract in exon 5 (c.3261dup/del) and poly A tract in exon 7 (c.3573del) were detected in several hypermutated samples and are not included in the table because they are presumed to be due to MSI.
