# Supplementary material for: Complex MSH2 and MSH6 mutations in hypermutated microsatellite unstable advanced prostate cancer
Source: Nat Commun. 2014 Sep 25;5:4988. doi: 10.1038/ncomms5988 (PMC4176888; doi:10.1038/ncomms5988)
Supplement: Supplementary Information — Supplementary Figures 1-14, Supplementary Tables 1-6 [file ncomms5988-s1.pdf]

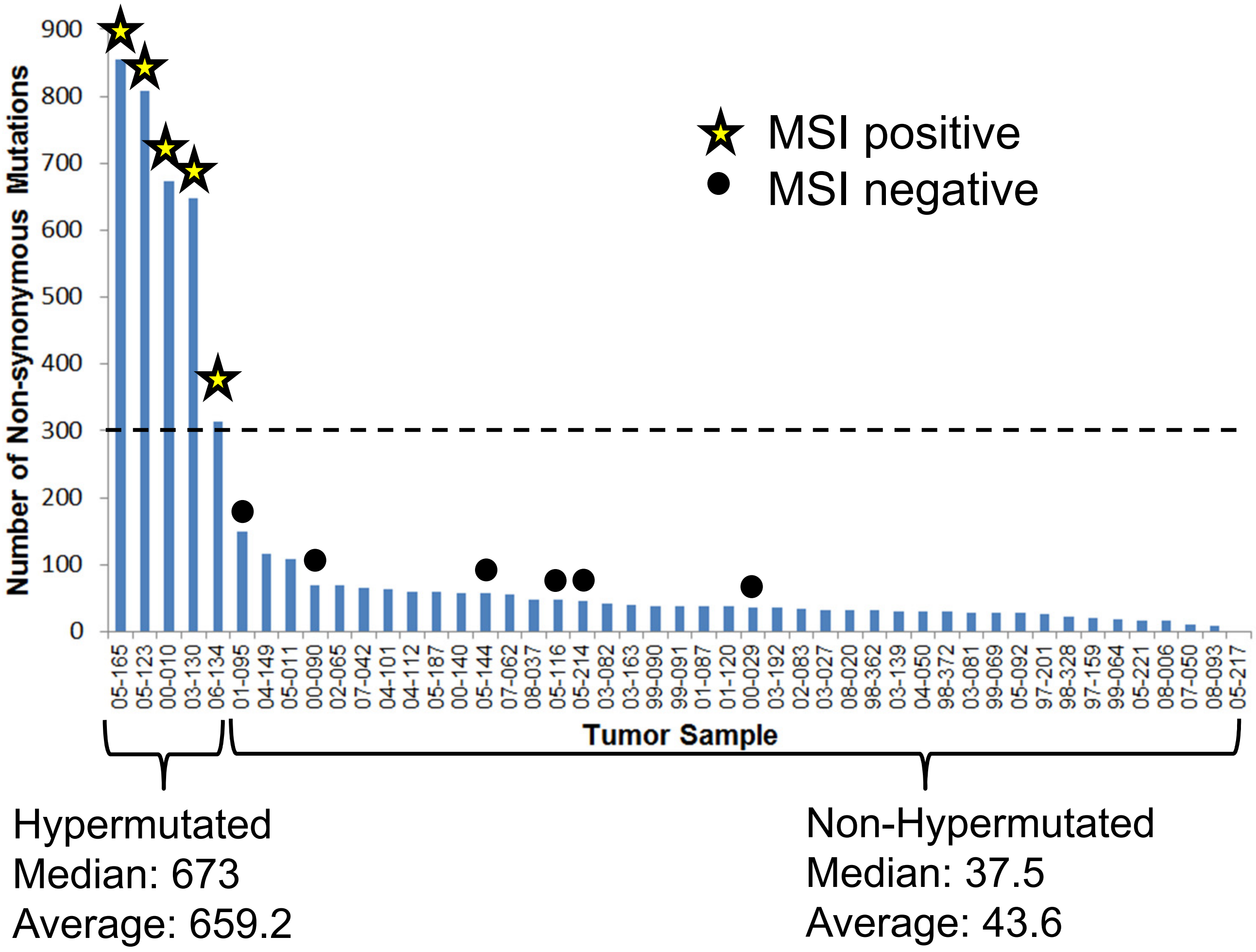

Supplementary Figure 1: Somatic Mutation Burden in Autopsy Cases by Exome Sequencing. Total number of somatic nonsynonymous mutations by exome sequencing for rapid autopsy cases. The threshold of 300 mutations used to determine hypermutation status is shown with the dashed line. Median and average mutation burden is given for both groups. Cases that had microsatellite instability testing are shown with yellow stars (positive) and black circles (negative).

LuCaP 58

1) *MSH6* del exon 8 through 3'UTR

2) *MSH6* frameshift (c.3799_3800del)

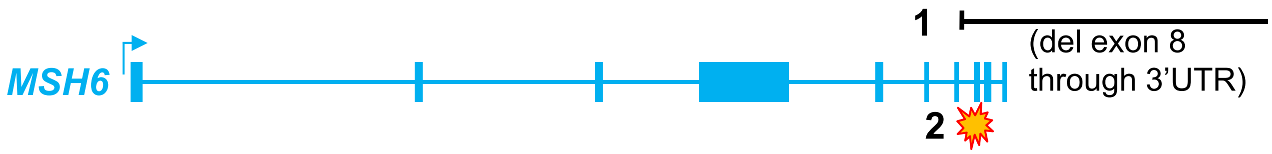

1)

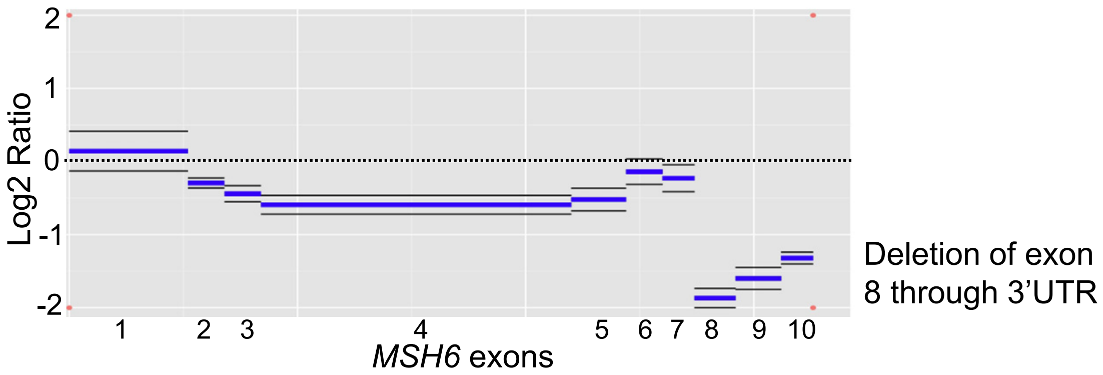

2) *MSH6* c.3799_3800del, p.M1267Gfs*7

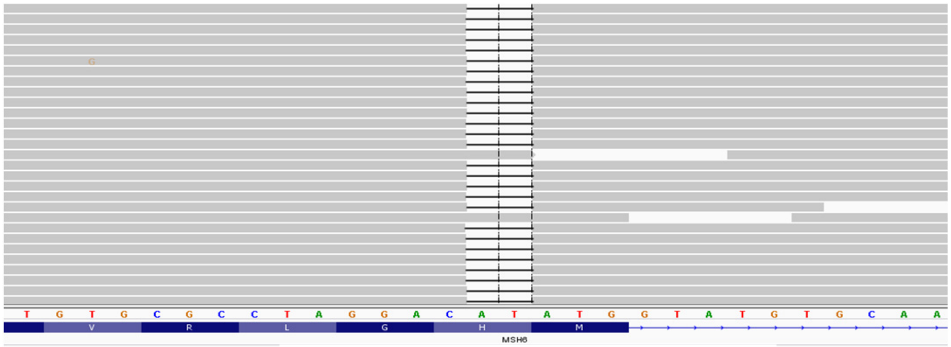

chr2:48033493-48033494delAT

Supplementary Figure 2: Detail on Mismatch Repair Gene Mutations in LuCaP 58. Two inactivating mutations were detected in *MSH6*. The first (1) is a deletion of exon 8 through the 3'UTR (top). Copy number was calculated from normalized depth of coverage of BROCA sequencing data and confirmed by genomic microarray (data not shown). The blue bars indicate exons 1-10 (from left to right) and black bars are the standard deviation of the measurement of Log2 ratio. The second (2) is a 2bp deletion resulting in a frameshift and premature truncation of the MSH6 protein (c.3799_3800del, bottom). Shown is a screenshot from the integrated genomics viewer of representative sequencing reads. The black bars indicated the deleted bases. The frameshift was detected in 314 out of a total of 360 sequencing reads, strongly supporting that there is bi-allelic inactivation of *MSH6*.

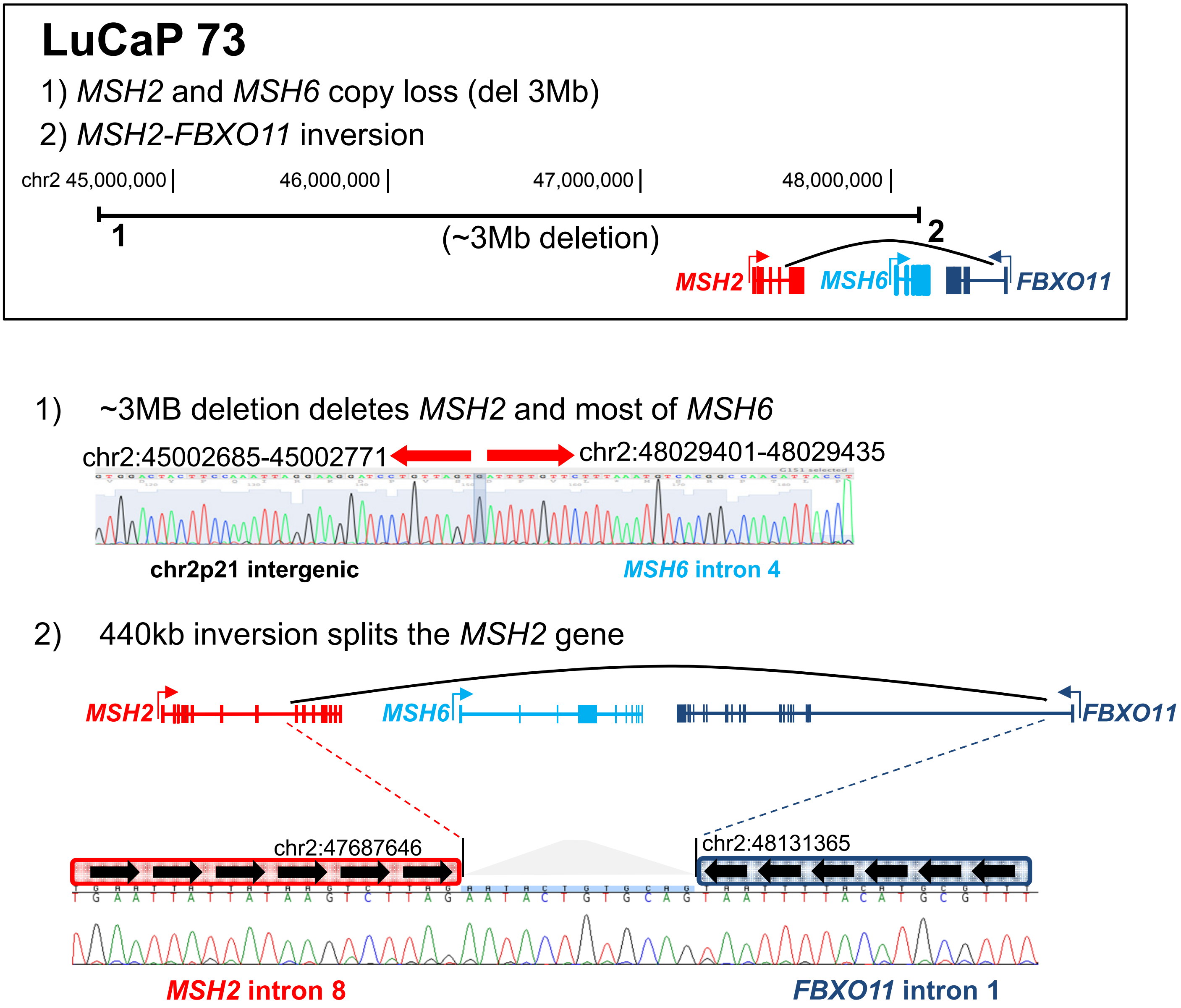

Supplementary Figure 3: Detail on Mismatch Repair Gene Mutations in LuCaP 73. Two large rearrangement mutations were detected at the *MSH2/MSH6* locus on chromosome 2 predicted to result in bi-allelic inactivation of *MSH2*. The first (1) is a 3Mb deletion that deletes both the MSH2 and MSH6 genes. The breakpoints where confirmed by Sanger sequencing as chr2:45,002,771-48,029,401 in hg19 genomic coordinates (top). The second (2) is a 440kb inversion mutation between *MSH2* intron 8 and *FBXO11* intron 1 that splits the *MSH2* gene and is predicted to result in loss of function. The breakpoints of the inversion were confirmed by Sanger sequencing as chr2:47687644-chr2:48131365 (bottom). There is a short inserted sequence between the two breakpoints.

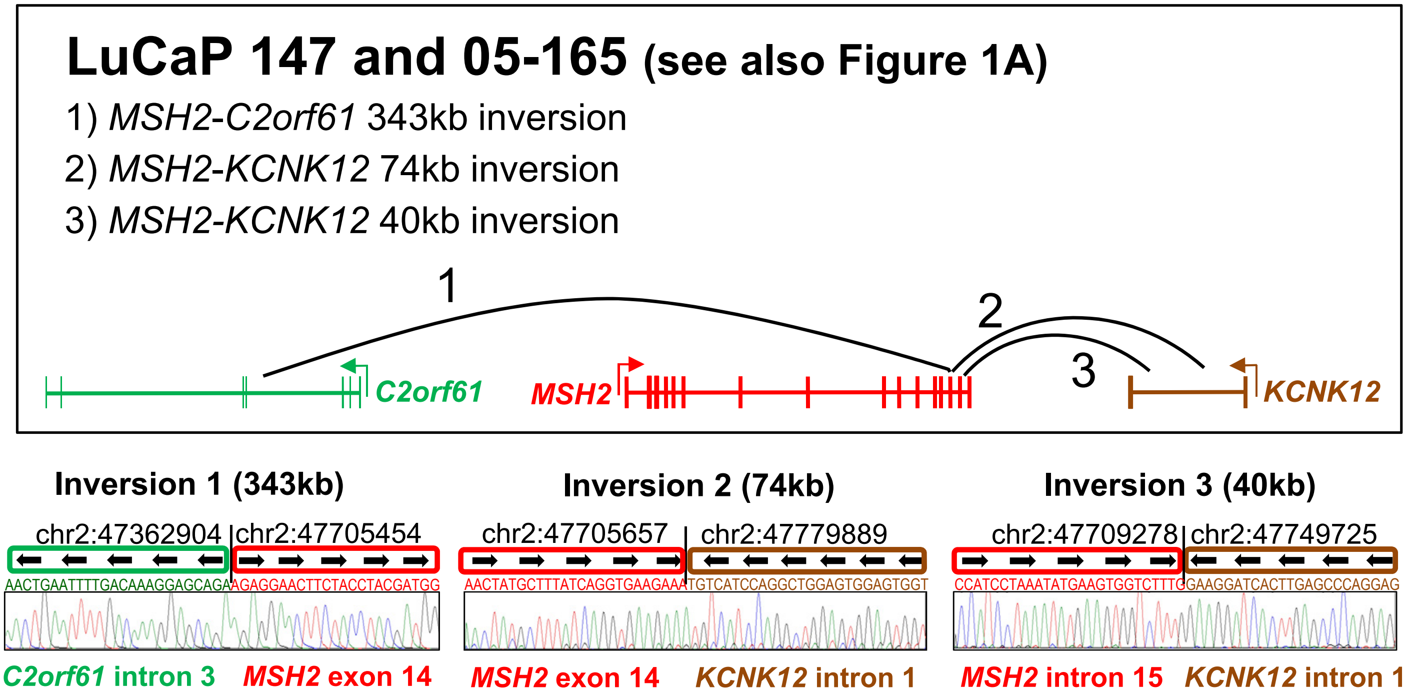

Supplementary Figure 4: Detail on Mismatch Repair Gene Mutations in LuCaP 147 and 05-165. Three different inversion mutations were detected involving the *MSH2* gene that are predicted to result in loss-of-function. The inversions were detected in all metastatic sites (bone, adrenal, liver, and lymph node). Each inversion was confirmed by Sanger sequencing with breakpoints given in hg19 genomic coordinates. LuCaP 147 was derived from autopsy patient 05-165 and the same mutations were detected in both, indicating that the *MSH2* structural rearrangements are not a result of xenografting.

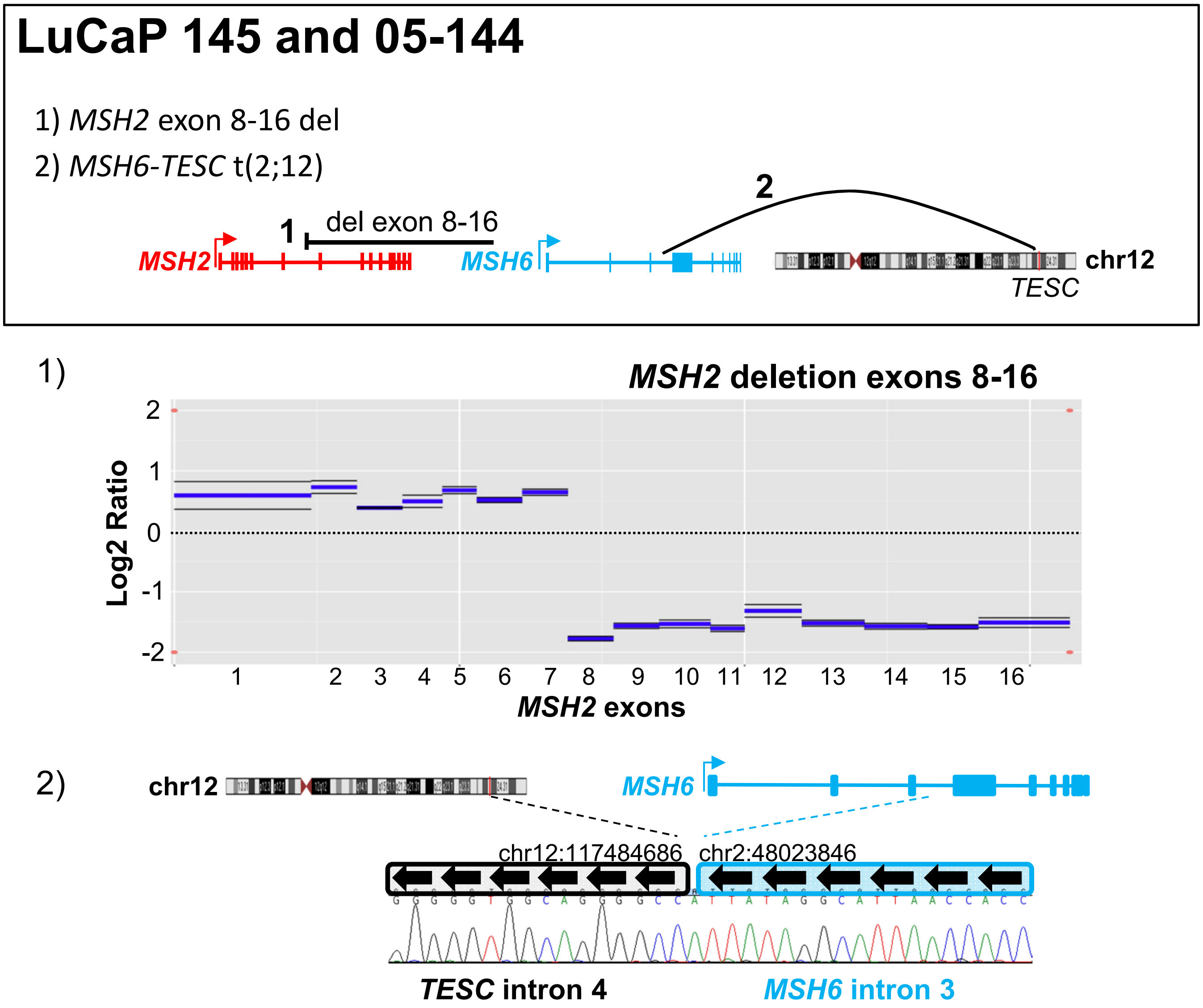

Supplementary Figure 5: Detail on Mismatch Repair Gene Mutations in LuCaP 145 and 05-144. Two mutations were detected. The first (1) is copy loss of exons 8-16 of *MSH2* (top). Copy number was calculated from normalized depth of coverage of BROCA sequencing data and confirmed by genomic microarray (data not shown). The blue bars indicate *MSH2* exons 1-16 (from left to right) and black bars are the standard deviation of the measurement of Log2 ratio. The second (2) is a translocation between *MSH6* intron 3 and *TESC* intron 4 on chromosome 12 q24.22. The breakpoints of the translocation were confirmed by Sanger sequencing. These tumors had neuroendocrine differentiation. Unlike the other tumors *MSH2/MSH6* rearrangements these tumors were not hypermutated and did not demonstrate MSI, most likely because one copy of *MSH2* and *MSH6* remain functionally intact. One hypothesis is that the cancer was ‘transitioning’ to a hypermutated state when the patient died, with a second hit in the *MSH2* or *MSH6* gene not yet acquired. LuCaP 145 was derived from autopsy patient 05-144 and the same mutations were detected in both, indicating that the structural rearrangements are not a result of xenografting.

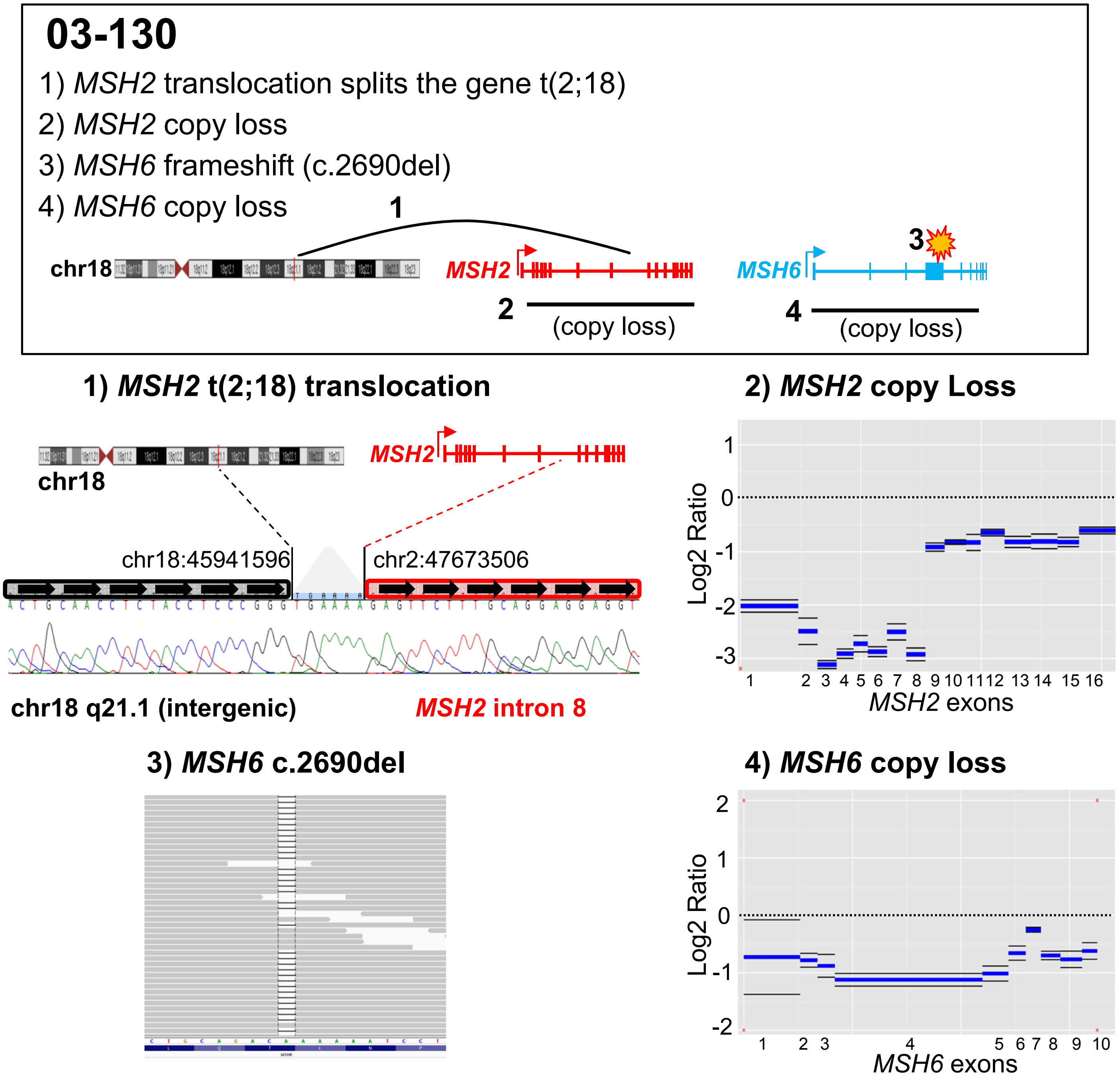

Supplementary Figure 6: Detail on Mismatch Repair Gene Mutations in 03-130. There was evidence of bi-allelic loss-of-function mutations in *MSH2* and *MSH6*. The first mutation (1) is a translocation between *MSH2* intron 8 *and* chr18 q21.1 (top left), in which the breakpoints were confirmed by Sanger sequencing. The second (2) is copy loss of *MSH2* (top right, homozygous in exons 1-8, likely as a result of the *MSH2* translocation). The third (3) is frameshift mutation in exon 4 of MSH6 (c.2690del, p.N897IfsX9). The black bars indicated the deleted bases. The frameshift was detected in 366 out of a total of 547 sequencing reads (67%), despite admixture of tumor with normal cells in the sample tested, supporting that there is bi-allelic inactivation of *MSH6* in tumor. The fourth (4) is copy loss of *MSH6*, probably single copy. In copy number plots blue bars indicate exons and black bars are the standard deviation of the measurement of Log2 ratio. Copy number was calculated by normalized depth of coverage of BROCA sequencing and confirmed by genomic microarray (data not shown)

06-134

MLH1 homozygous copy loss

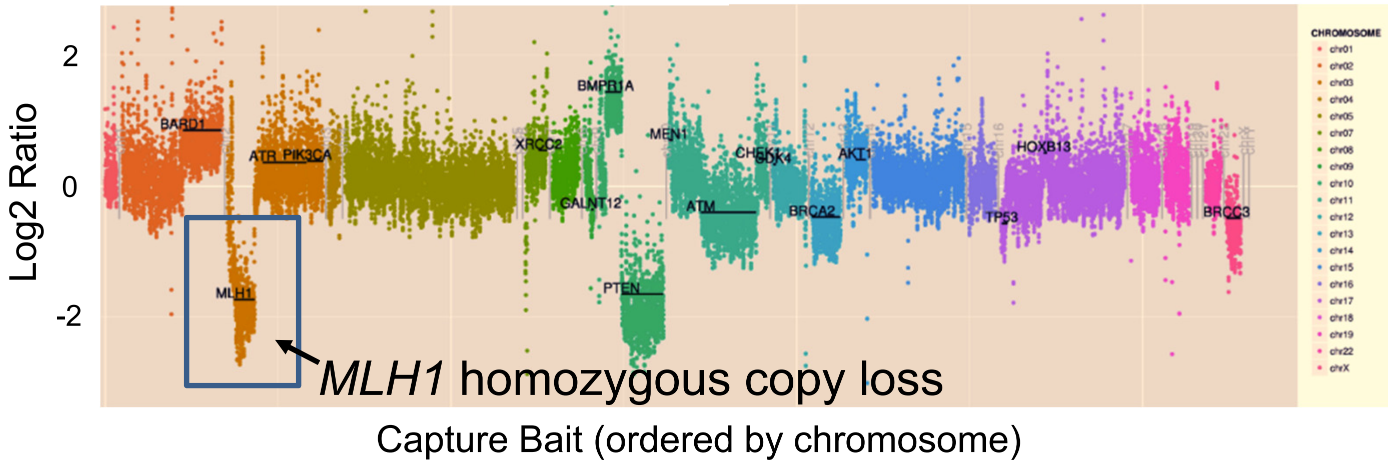

Supplementary Figure 7: Detail on Mismatch Repair Gene Mutations in 06-134. The sample tested had *MLH1* copy loss which is very likely to be homozygous and result in complete loss of MLH1 protein function. Depicted is copy number analysis by BROCA targeted deep sequencing. *MLH1* deletion was confirmed by genomic microarray (data not shown). The Log2 ratio in relationship to a normal female control patient. *BRCC3* (far right) on the X chromosome can be used to calibrate the Log2 ratio expected with heterozygous copy loss because this is a male patient. *PTEN* is also deleted in this patient's tumor, a common event in metastatic prostate cancer.

00-010

MSH2 frameshift (c.2364_2365insTACA, p.A789YfsX11)

hg19 chr2:47705564 insert TACA

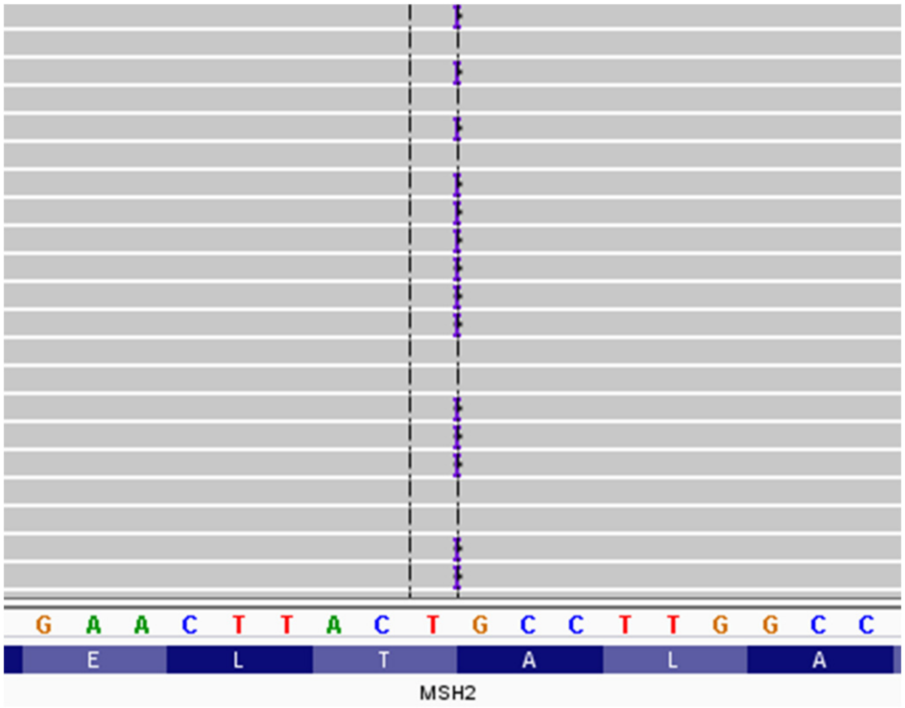

Supplementary Figure 8: Detail on Mismatch Repair Gene Mutations in 00-010. The primary prostate and liver tumor metastasis samples tested had a frameshift loss-of-function mutation in *MSH2* (c.2364_2365insTACA, p.A789YfsX11). The purple "I" indicates the position of the inserted bases, visualized in the integrated genomics viewer. A second loss-of-function mutation was not detected. It is suspected a second *MSH2* loss-of-function mutation is present because the case was MSI high, had loss of MSH2 and MSH6 protein by IHC, was *MLH1* unmethylated, and had intact MLH1 protein IHC (see separate figures).

05-0123

1) *MSH2* frameshift (c.1124_1125insG)
2) *MSH2* frameshift (c.1082del)
3) *MLH1* frameshift (c.1310del), lymph node only

MSH2 c.1082del, p.N361IfsX2
chr2:47656885delA

MSH2 c.1124_1125insG, p.L376FfsX13
chr2:47656928insG

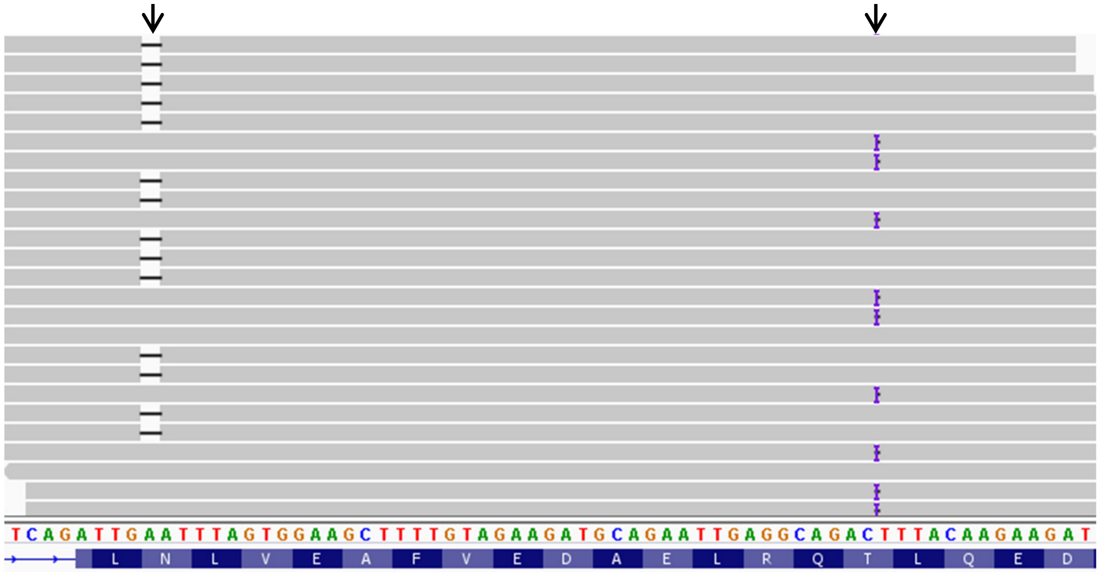

MSH2 exon 7

chr3:37067396delC

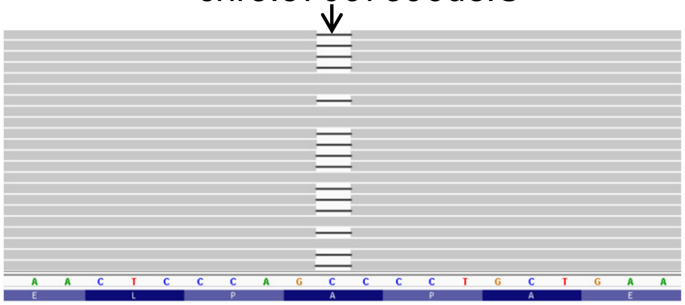

MLH1 c.1310del, p.P437LfsX54
(Lymph node metastasis only)

MLH1 exon 12

Supplementary Figure 9: Detail on Mismatch Repair Gene Mutations in 05-123. The primary prostate and lymph node metastasis tumor samples tested had bi-allelic frameshift loss-of-function mutations in *MSH2* exon 7 (c.1082del and c.1124_1125insG, top). The black bars indicate sequencing reads with the c.1082del deletion; the purple "I" indicates the position of the inserted bases in the c.1124_1125insertion, visualized in the integrated genomics viewer. Note that these two mutations do not occur on sequencing reads from the same allele, strongly supporting that they are *in trans (*bi-allelic). In addition, an *MLH1* frameshift mutation in exon 12 (c.1310delC) was detected in the lymph node metastasis sample only (bottom).

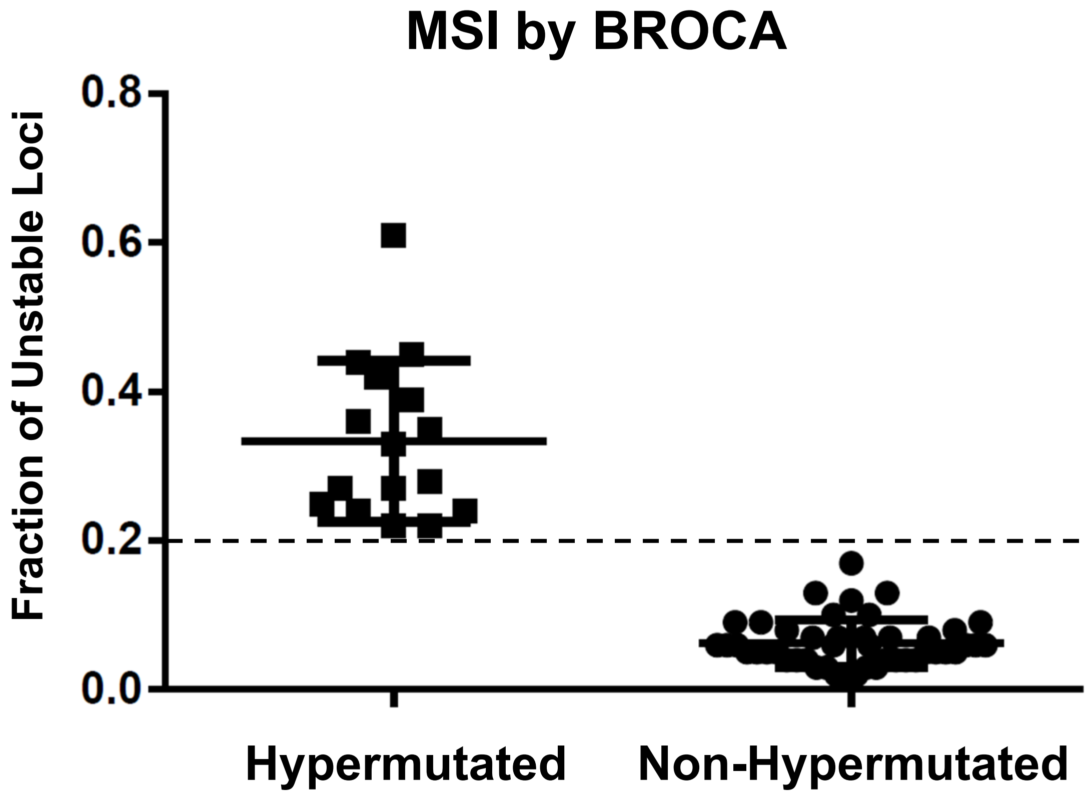

Supplementary Figure 10: Microsatellite Instability Results by BROCA Next-Generation Sequencing. We developed an approach to measure microsatellite instability directly from next-generation sequencing data that we call mSINGS. This method is described in the methods section (Salipante et al. 2014 *Clinical Chemistry*, in press). The fraction of unstable microsatellite loci out of a maximum of 146 mononucleotide microsatellite loci captured by BROCA is given on the y-axis. A threshold of 0.2 (20%) unstable loci was established as a cutoff for microsatellite instability (dashed line). Solid lines represent the median fraction of unstable loci for hypermutated and non-hyermutated cases. The raw data used to generate this summary figure, including which loci were unstable by BROCA in each sample is given in Supplementary Data 3.

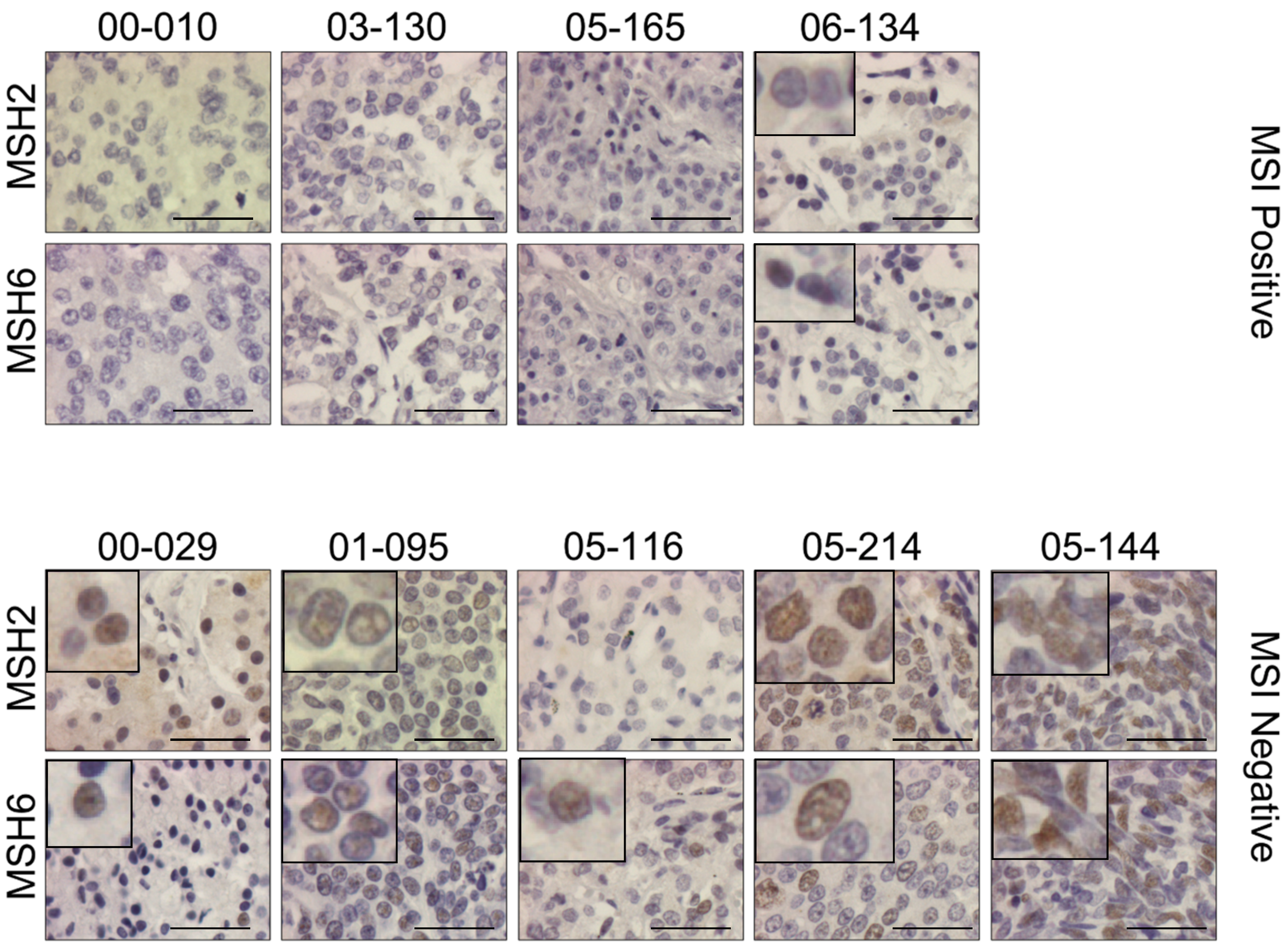

Supplementary Figure 11: IHC results for prostate rapid autopsy metastasis samples. Hypermutated MSI positive autopsy cases 00-010, 03-130, 05-165, which harbored somatic mutations in *MSH2*, *MSH6* or both genes show complete loss of MSH2 and MSH6 expression by IHC using a tissue microarray (top panels). Tissue was not available for IHC studies in hypermutated case 05-123. Hypermutated MSI positive case 06-134, which had somatic deletion of *MLH1*, has focal intact nuclear expression of MSH2 and MSH6 protein (top left, insets). By contrast, MSH2 and MSH6 nuclear staining is intact in MSI-negative autopsy cases 00-029, 01-095, 05-214, and 05-144 (bottom panels, examples of positive nuclear staining in insets). MSH6, but not MSH2 protein expression was detected MSI-negative case 05-116, a case that also had absent MLH1 protein (see separate figure). This could reflect a false negative result due to poor quality tissue for this sample on the tissue microarray. For MSH2 and MSH6, heterogeneity of immunostaining is common in tumor tissue, and protein expression is generally considered intact if any cells display positive nuclear staining. Because MSH2 and MSH6 function as a heterodimer, mutations in one gene frequently result in loss of expression of both proteins, particularly when there are *MSH2* mutations. All samples are from metastases and not primary tumors. Scale bar: 0.1mm.

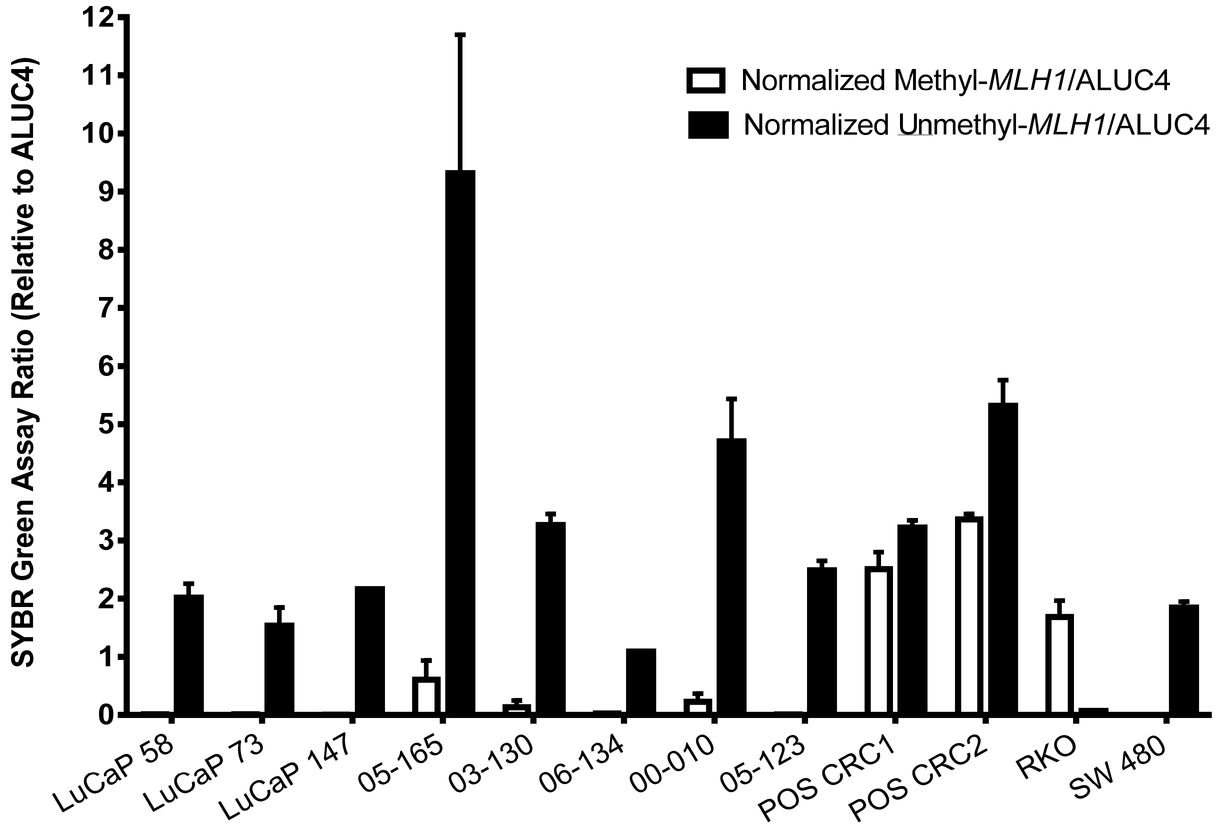

Sample	*MLH1* methylation status
LuCaP 58	Unmethylated
LuCaP 73	Unmethylated
LuCaP 147	Unmethylated
05-165-K3	Unmethylated
03-130-L2	Unmethylated
06-134-P1	Unmethylated
00-010	Unmethylated
05-123-D1	Unmethylated
POS CRC 1 (known methylated colon tumor)	Methylated
POS CRC 2 (known methylated colon tumor)	Methylated
RKO (positive control cell line)	Methylated
SW 480 (negative control cell line)	Unmethylated

Supplementary Figure 12: Hypermutated Prostate Tumors Do Not Exhibit *MLH1* Methylation. Results of a *MLH1* methylation-specific SYBR green assay are expressed as ratios between Methyl-MLH1 or Unmethyl-MLH1 values and the ALUC4 control values. Genomic DNA samples were bisulfite-treated with EZ DNA Methylation Kit (Zymo Research, Irvine, CA) according to manufacturer's protocol. The primers used in the SYBR Green Assay were previously described (see methods). The error bars represent the standard error of the mean. DNA samples from 2 known *MLH1* methylated colon cancer tumors (POS CRC1 and POS CRC2) and cancer cell lines RKO (methylated) and SW480 (unmethylated) are used as controls for the assay.

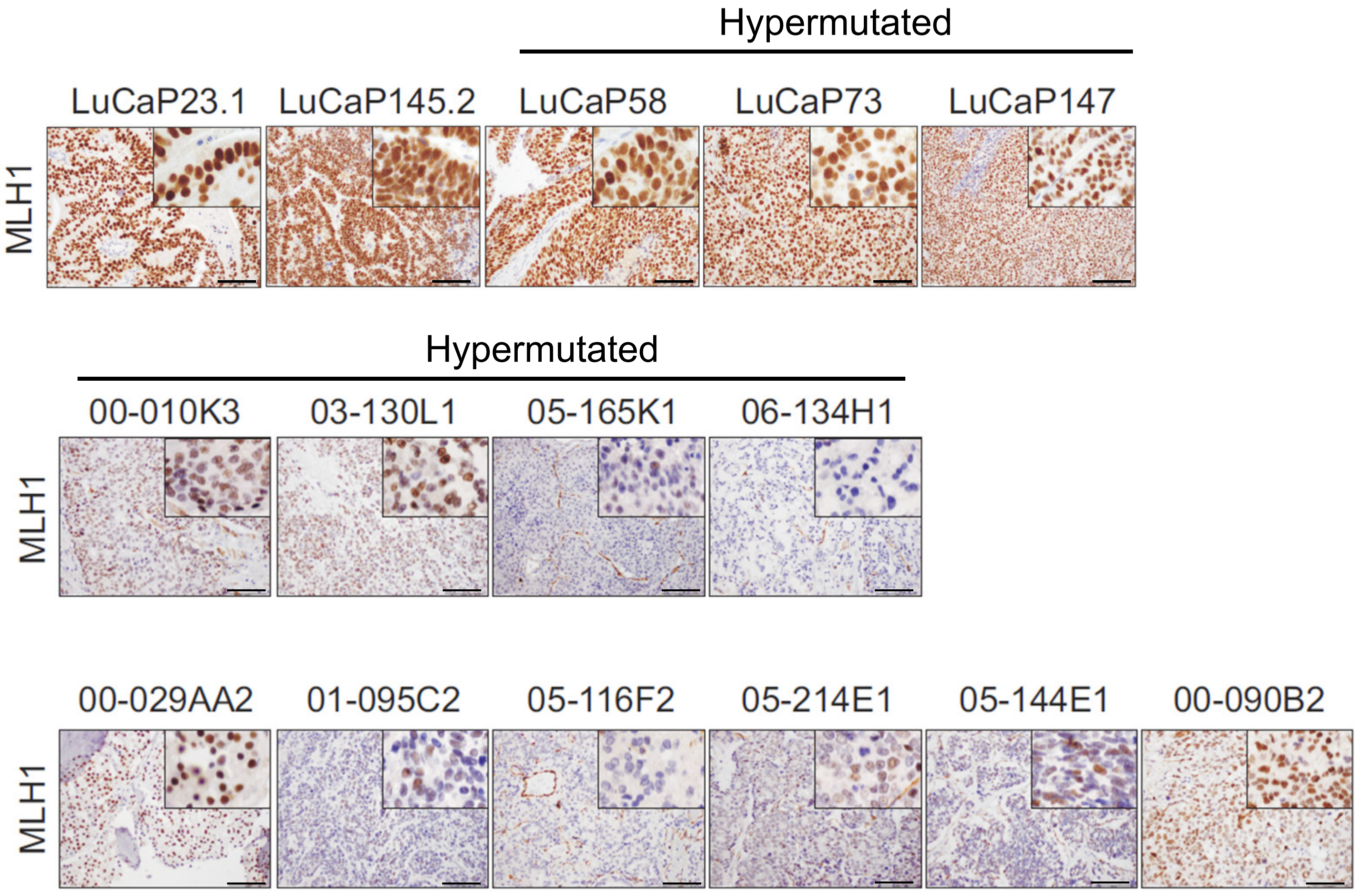

Supplementary Figure 13: Hypermutated MSI Positive Prostate Tumors With *MSH2* or *MSH6* mutations have intact MLH1 protein by IHC. Hypermutated MSI positive cases LuCaP 58, LuCaP 73, LuCaP 147 (top) and autopsy cases 00-010, 03-130, 05-165 (middle), which harbored somatic mutations in *MSH2*, *MSH6* or both have intact MLH1 expression by IHC using a tissue microarray. This corroborates the *MLH1* methylation studies and strongly argues against *MLH1* epigenetic silencing as a mechanism of MSI in these tumors. Hypermutated MSI positive case 06-134 that had homozygous deletion of *MLH1* has absent MLH1 protein. Tissue was not available for IHC studies in hypermutated case 05-123. MLH1 protein expression was not detected MSI-negative case 05-116 (bottom), a case that also had absent MSH2 protein (see separate figure). This could reflect a false negative result due to poor quality tissue for this sample on the tissue microarray. For MLH1, heterogeneity of immunostaining is common in tumor tissue, and protein expression is generally considered intact if any cells display positive nuclear staining. Scale bar: 0.1mm.

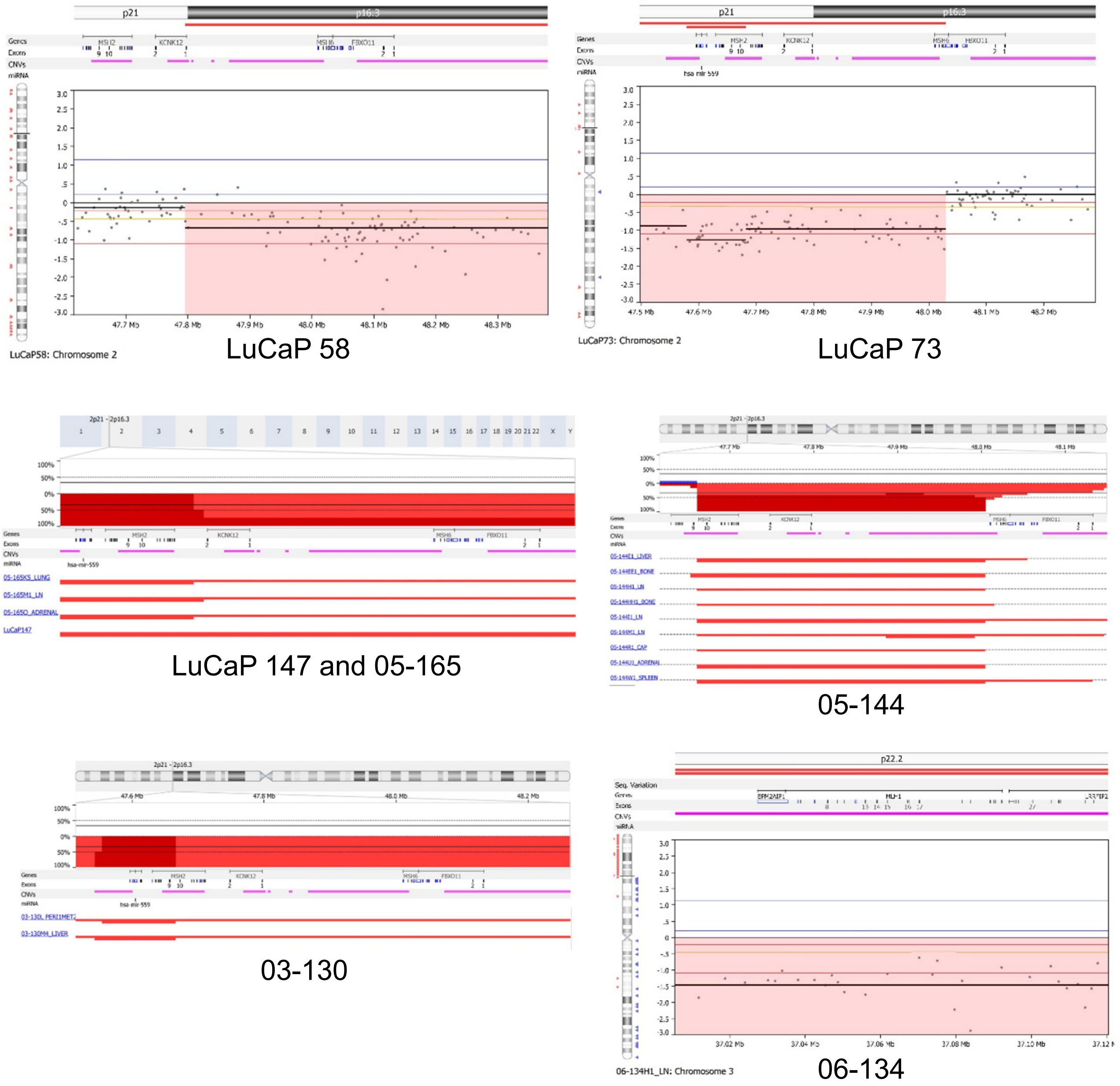

Supplementary Figure 14: Confirmation of *MSH2*, *MSH6*, and *MLH1* Copy Number Status by Genomic Microarray. Genomic microarray (array CGH) was performed for all cases that had *MSH2* and *MSH6* structural rearrangements and also for case 06-134 with *MLH1* homozygous gene deletion. The genomic loci are given along the top of each panel. Y axes are log2 ratio compared to normal control. Red indicates deletion. The results are concordant with copy number status as assessed by BROCA next-generation sequencing.

Supplementary Table 1: Patients

Unique Patient Number*	LuCaP ID	Autopsy Patient ID	Autopsy Sample Exome	Autopsy Site	Number of germline and somatic protein altering variants**	Number of somatic protein altering mutations***	Hyper-mutated?	BROCA deep sequencing
1	LuCaP 23.1				643			X
2	LuCaP 35,35CR				179 and 203			X
3	LuCaP 58				4067		X	X
4	LuCaP 70				389			X
5	LuCaP 73				2972		X	X
6	LuCaP 77				451			X
7	LuCaP 78	98-328	98-328-J	lymph node	372	23		X (LuCaP only)
8	LuCaP 81	98-362	98-362-B1	lymph node	304	32		X (LuCaP only)
9	LuCaP 86.2				326			X
10	LuCaP 92	99-069	99-069-G1	perito-neal	313	29		X (LuCaP only)
11	LuCaP 96,96CR				282 and 240			X
12	LuCaP105				374			X
13	LuCaP 141				701		X	X
14	LuCaP 145.1/145.2	05-144	05-144-M1	lymph node	538	57		X (LuCaP and Autopsy)
15	LuCaP 147	05-165	05-165-K5	lung	2714	855	X	X (LuCaP and Autopsy)
16		00-010	00-010-K	liver		673	X	X
17		00-029	00-029-N9	lymph node		37		X
18		00-090	00-090-T3	lymph node		69		X
19		00-140	00-140-J9	lymph node		58		
20		01-087	01-087-MM	bone		38		
21		01-095	01-095-N1	lymph node		149		X
22		01-120	01-120-A1	liver		38		
23		01-181	NA	NA		NA		
24		02-065	02-065-N1	skin		69		
25		02-083	02-083-E1	lymph node		34		
26		03-027	03-027-M1	lung		33		
27		03-081	03-081-L	lymph node		29		
28		03-082	03-082-H1	liver		42		
29		03-130	03-130-M4	liver		647	X	X
30		03-139	03-139-M9	retro-perito-neal		31		
31		03-163	03-163-S4	liver		40		
32		03-192	03-192-B3	lung		37		
33		04-050	04-050-W1	lymph node		30		

34		04-101	04-101-H1	perito-neal		63		
35		04-112	04-112-M1	lymph node		60		
36		04-149	04-149-E2	lymph node		117		
37		05-011	05-011-D2	lymph node		108		
38		05-092	05-092-E7	liver		28		
39		05-116	05-116-F	lung		47		X
40		05-123	05-123-E2	lymph node		807	X	X
41		05-187	05-187-J3	lymph node		59		
42		05-214	05-214-G1	lymph node		46		X
43		05-217	05-217-L3	lung		2		
44		05-221	05-221-MM1	bone		17		
45		06-047	NA	NA		NA		
46		06-081	NA	NA		NA		
47		06-134	06-134-H1	lymph node		314	X	X
48		07-042	07-042-H2	lymph node		65		
49		07-050	07-050-EE1	bone		11		
50		07-062	07-062-F2	lung		55		
51		08-006	08-006-H1	lymph node		16		
52		08-020	08-020-N2	lymph node		32		
53		08-037	08-037-F1	liver		48		
54		08-093	08-093-J1	lymph node		9		
55		97-159	97-159-H2	liver		20		
56		97-201	97-201-K	lymph node		27		
57		98-372	98-372-L	lymph node		30		
58		99-064	99-064-O	lymph node		19		
59		99-090	99-090-A2	liver		39		
60		99-091	99-091-C	liver		39		

*There was partial overlap of LuCaP and autopsy patients as indicated by samples with both LuCaP ID and Autopsy ID.
**After subtracting against databases of germline variants (LuCaP exome without matched normal). Numbers are from Kumar et al. PNAS 2011 108(41):17087-92. Note that LuCaP 35CR = LuCaP 35V, LuCaP 96CR= LuCaP 96AI.
***From a representative metastasis (autopsy patient exome with matched normal). Differences in mutation counts between autopsy metastases and LuCaP xenografts are attributable to germline variants included in LuCaP
NA refers to cases where the corresponding normal was not available

Supplementary Table 2: Targeted Capture Gene List (BROCA assay)

DNA Repair Related	*ATM*	*ATR*	*BAP1*	*BARD1*	*BRCA1*	*BRCA2*	*BRCC3*	*BRIP1*
	CHEK1	*CHEK2*	*FAM175A*	***MLH1***	*MRE11A*	***MSH2***	***MSH6***	*NBN*
	PALB2	***PMS2***	*PRSS1*	*PTEN*	*RAD50*	*RAD51B*	*RAD51C*	*RAD51D*
	RBBP8	*TP53*	*TP53BP1*	*XRCC2*				
Additional Cancer-Related	*AKT1*	*APC*	*BMPR1A*	*CDH1*	*CDK4*	*CDKN2A*	*CTNNA1*	*GALNT12*
	GEN1	*GREM1*	*HOXB13*	*MEN1*	*MUTYH*	*PIK3CA*	*POLD1*	*POLE*
	PPM1D	*RET*	*SDHB*	*SDHC*	*SDHD*	*SMAD4*	*STK11*	*VHL*

*Mismatch DNA repair genes in BOLD

Supplementary Table 3: Mutations in Additional DNA Repair Genes on BROCA

Case*	Hyper-mutated?	ATM	ATR	BARD1	BRCA1	BRCA2	BRIP1	CHEK1	CHEK2	FAM175A	MRE11A	NBN	PALB2	RAD51C	RAD51D	TP53BP1	XRCC2
05-165	Yes	FS/MS				FS											
03-130	Yes				SR												
06-134	Yes	FS/CL		MS		CL											
00-010	Yes					CL					FS						
05-123	Yes	FS			CL	FS/CL											
01-095	No					CL/CL			MS								
05-144	No	MS/MS				CL			CL								
05-214	No					CL			CL								CL
05-116	No	SR									SR						
00-029	No				CL/CL												
00-090	No																
LuCaP 58	Yes	MS			SR	MS		MS				MS					
LuCaP 73	Yes	MS				FS/MS	MS				FS/SP				FS	FS	MS
LuCaP 147	Yes	FS/MS				FS										FS	
LuCaP 23.1	No			CL		CL											
LuCaP 35	No		CL														
LuCaP 70	No																
LuCaP 77	No													SR			
LuCaP 78	No	CL		CL							CL						SR
LuCaP 81	No	CL															
LuCaP 86.2	No		CL			CL											
LuCaP 92	No								CL								
LuCaP 96	No					FS/NS			SR								
LuCaP 105	No					CL			CL								
LuCaP 141	No				MS				CL	CL							
LuCaP 145.1,145.2	No	MS/MS				CL			CL								

Key: CL= copy loss, SR= structural rearrangement, MS= missense mutation, FS= frameshift mutation, NS= nonsense mutation, SP= splice site mutation

*For patients in which multiple tumor sites were sequenced, mutations were detected in all sites

Supplementary Table 4: Antibodies used in IHC

Primary Antibody	Company	Clone	Dilution
MLH1	Cell Marque	G168-728	1:50
MSH2	Cell Marque	G219-1129	1:100
MSH6	Biocare Medical	BC/44	1:50
PMS2	Cell Marque	ERP3947	1:04

Supplementary Table 5: Primers used in confirmation of somatic rearrangements

Rearrangement	Sample	Primer1	Primer2
MSH2 Translocation	03-130 (block L2)	TTAAAATGTGTCACAAAATTCATGG	TTCGACAACTATTTAGGAGATGCAC
MSH2 Inversion v2c (9278)	LuCap 147	CTGTTGTACCTATACAAGCAGGAGA	CTTTGGTCAAACGATCATATTTTCT
MSH2 Inversion v2a (5660)	LuCap 147	GGCCAATCAGATACCAACTGTTAAT	AATCTCCAGATGCATTGTTAGAAAC
MSH2 Inversion v2b (5452)	LuCap 147	GCAAAGTTAGAGGACTGACACTACC	ACCAATCTTTGTTGCAATGTATTCT
MSH2 Rearrangement	LuCap 73	CTGAAGACGCCCAGTTTCCT	GCACAAGATCTCCAACTGCC
MSH6 Translocation	05-144 (block LL2)	ACCTTGGCTCAAACGTTAATGG	ATCAGAAGCACTGTGGACCG

Supplementary Table 6: Clinical Data for Autopsy Patients Tested by BROCA

Rapid Autopsy Patient	Hypermutated?	Gleason Score	Age at Diagnosis	Age at Death (years)	Survival after Diagnosis (years)	Final Serum PSA (ng/mL)	Androgen Ablation	Castration Resistance (years)	Ketaconazole	Diethylstilbestrol	Corticosteroid	Taxotere	Carboplatin	Estrmustine	Age at First Bone Metastasis (years)	Survival from First Bone Metastasis (years)	Bisphosphonates	Bisphosphonate Duration (years)
05-165	Y	7	67	69	2.2	96.2	Y	1.2	Y	Y	Y	Y	N	N	67	1.8	Y	1.7
03-130	Y	8	63	65	1.6	106.1	Y	1.5	N	N	Y	Y	N	Y	63	1.6	Y	1.5
06-134	Y	9	67	73	5.9	55.6	Y	2.5	Y	Y	Y	Y	Y	N	72	0.7	Y	0.4
00-010	Y	7	80	84	4.3	128.7	Y	2.4	N	N	Y	N	N	N	80	4.3	Y	1.0
05-123	Y	clinical data not available																
00-029	N	9	77	84	6.9	2038.9	Y	1.1	N	N	N	N	N	N	81	2.9	Y	0.5
00-090	N	8	66	70	4.5	325.0	Y	3.4	N	Y	N	Y	Y	Y	70	0.4	Y	1.8
01-095	N	7	62	72	9.6	105.6	Y	4.9	N	Y	Y	Y	N	Y	67	4.8	N	-
05-116	N	8	63	80	17.9	122.8	Y	7.7	Y	N	Y	Y	N	Y	75	5.4	Y	4.9
05-144	N	9	59	62	2.4	0.2	Y	0.6	Y	N	Y	N	N	N	59	2.4	Y	0.1
05-214	N	6	62	70	7.7	2017.6	Y	3.2	N	N	Y	Y	Y	N	68	1.6	Y	0.8
